# Prevalence, component patterns, and lifestyle correlates of metabolic syndrome among civil servants undergoing routine health examinations in Guangzhou, China: a cross-sectional study

**DOI:** 10.3389/fpubh.2026.1893244

**Published:** 2026-07-06

**Authors:** Wenzhuo Feng, Yue Li, Xiaoliang Guo, Jiakang Chen, Wenwei Luo, Weizheng Zhang

**Affiliations:** 1 Clinical Laboratory, Guangzhou Cadre and Talent Health Management Center, Guangzhou Eleventh People's Hospital, Guangzhou, Guangdong, China; 2 Medical Record Department, Guangzhou Cadre and Talent Health Management Center, Guangzhou Eleventh People's Hospital, Guangzhou, Guangdong, China; 3 Department of Critical Care Medicine, The First Affiliated Hospital of Guangdong Pharmaceutical University, Guangzhou, Guangdong, China

**Keywords:** cross-sectional study, metabolic syndrome, occupational health, physical activity, urban health

## Abstract

**Introduction:**

Metabolic syndrome (MetS) is an important cardiometabolic risk condition among working-age adults, but evidence on its burden, component patterns, and lifestyle correlates among office-based occupational populations in southern China remains limited.

**Methods:**

This cross-sectional study identified 22,156 adults undergoing routine health examinations in Guangzhou, China, in 2023. Primary descriptive and multivariable analyses were conducted among 11,291 participants with complete biochemical, anthropometric, and questionnaire data. MetS was defined according to the International Diabetes Federation 2005 criteria. Multiple imputation by chained equations was performed as a sensitivity analysis using the full eligible cohort, and a fully adjusted BMI-adjusted model was additionally fitted within the imputed datasets.

**Results:**

In the complete-case sample, MetS prevalence was 13.8%. Elevated blood pressure was the most frequent abnormal component, followed by elevated triglycerides, elevated fasting glucose, central obesity, and reduced HDL-C. In the primary complete-case multivariable analysis, sufficient physical activity was inversely associated with MetS (OR = 0.85, 95% CI: 0.75–0.96). This association was consistent in the multiple-imputation sensitivity analysis (OR = 0.80, 95% CI: 0.72–0.89) and in the fully adjusted BMI-adjusted sensitivity model (OR = 0.80, 95% CI: 0.70–0.91).

**Discussion:**

MetS was common in this occupational routine-examination cohort. Elevated blood pressure was the most frequent abnormal component, and physical activity was the most consistent lifestyle correlate. These findings support workplace-based physical activity promotion and cardiometabolic risk management as potential priorities for similar office-based occupational populations.

## 1 Introduction

Metabolic syndrome (MetS) is a cluster of metabolic abnormalities characterized by central obesity, dyslipidemia, elevated blood pressure, and impaired glucose metabolism, and it substantially elevates the risks of cardiovascular disease, type 2 diabetes, and premature mortality. Globally, an estimated 20–30% of adults are affected by MetS, underscoring a major and persistent public-health burden (1, 2).

In China, MetS prevalence has risen in parallel with rapid urbanization and lifestyle transitions. Pooled estimates indicate that roughly one-quarter of Chinese adults meet diagnostic criteria for MetS, though absolute rates vary with definitions and survey years (3–5). Recent national and regional investigations further show heterogeneity across sex, age, and urban–rural strata, with higher prevalence generally in urban residents, men, and older adults (3–6).

Guangzhou, a major metropolis in southern China, has experienced pronounced dietary shifts toward energy-dense foods, reduced physical activity, and sedentary work styles—all factors linked to MetS (7–9). Notably, office-based and administrative workers (e.g., civil servants) often face prolonged sedentary time and occupational stress, factors associated with greater MetS risk independent of leisure-time physical activity (8, 9). Despite this, evidence specific to civil servants and other office-based professional groups in southern China remains limited. Routine health examination cohorts in occupational settings provide real-world opportunities to characterize cardiometabolic risk profiles and identify modifiable lifestyle correlates that may inform workplace health promotion and occupational health management. Therefore, this study aimed to estimate the prevalence and component distribution of MetS and to examine demographic and lifestyle correlates of MetS among civil servants undergoing routine health examinations in Guangzhou, China.

## 2 Materials and methods

### 2.1 Data source

This cross-sectional study was conducted at a routine health examination center serving civil servants and professional workers in Guangzhou, China, in 2023. A total of 22,156 eligible adults who underwent routine physical examinations were identified. Of these, 11,291 participants with complete biochemical, anthropometric, and questionnaire data were included in the primary complete-case analyses. Among all eligible participants, 10,865 (49.0%) had incomplete biochemical and/or questionnaire data. Compared with included participants, excluded eligible examinees were older and more likely to be female and had substantial missingness in questionnaire-based socioeconomic and lifestyle variables (Supplementary Table S1). Multiple-imputation analyses using the full eligible cohort were performed as sensitivity analyses to evaluate the influence of missing data.

### 2.2 Data collection

Demographic and lifestyle information, including age, sex, smoking status, alcohol consumption, dietary behaviors, physical activity, sedentary behavior, sun exposure, tea consumption, and sleep duration, was obtained through standardized electronic questionnaires. Medical history information, including histories of hypertension, diabetes, and dyslipidemia, was obtained from routine health examination medical history records. Anthropometric measurements, including height, weight, waist circumference, hip circumference, systolic blood pressure, and diastolic blood pressure, were performed by trained staff using calibrated instruments. Laboratory tests were conducted after an overnight fast and included fasting plasma glucose (FPG), triglycerides (TG), high-density lipoprotein cholesterol (HDL-C), total cholesterol (TC), and low-density lipoprotein cholesterol (LDL-C). Body mass index (BMI) was calculated as weight in kilograms divided by height in meters squared (kg/m^2^).

Routine physical examinations were conducted according to the standardized health examination workflow of the center. Participants first completed the electronic questionnaire before the clinical examination. Trained staff then performed anthropometric and blood pressure measurements using calibrated instruments, and venous blood samples were collected after an overnight fast for biochemical testing. Details of questionnaire-derived variables, including item content, original response options, recoding or unit conversion, and analytic coding, are provided in Supplementary Table S5. Questionnaire response options were retained in their original form in Supplementary Table S5, and any category collapsing or unit conversion used for the present analysis is explicitly described. Most questionnaire variables were single-item or item-group screening questions rather than multi-item psychometric scales; therefore, subscale scores and internal consistency measures such as Cronbach’s alpha were not applicable.

### 2.3 Definition of MetS and its components

MetS was defined according to the International Diabetes Federation (IDF) 2005 criteria2. Central obesity (waist circumference ≥90 cm for men and ≥80 cm for women, based on Chinese population cutoffs) was considered a prerequisite. In addition, the presence of two or more of the following four components confirmed the diagnosis of MetS:

1. Elevated triglycerides (TG ≥ 1.7 mmol/L, or specific treatment for this condition);
2. Reduced HDL-C (<1.03 mmol/L in men, <1.29 mmol/L in women, or specific treatment);
3. Elevated blood pressure (systolic BP ≥ 130 mmHg, diastolic BP ≥ 85 mmHg, or treatment of diagnosed hypertension);
4. Elevated fasting plasma glucose (FPG ≥ 5.6 mmol/L, or previously diagnosed type 2 diabetes).

### 2.4 Covariates

Candidate demographic and lifestyle covariates were collected via standardized electronic questionnaires and anthropometric measurements. Variables considered in descriptive and regression analyses are described below:

1. Body Mass Index (BMI): calculated as weight in kilograms divided by height in meters squared(kg/m^2^). BMI was categorized as normal (18.5 ≤ BMI < 24 kg/m^2^), overweight (24 ≤ BMI < 28 kg/m^2^), or obese (≥28 kg/m^2^).
2. Education Level: categorized as low (junior school or below), moderate (high school), or high (Bachelor’s degree or above).
3. Annual Household Income: categorized into quartiles as low, moderate, high, or very high.
4. Smoking Status: categorized as never smoking or current/former smoking, with current/former smoking defined as a reported smoking history of ≥1 year.
5. Alcohol Consumption: categorized as non-drinking or current drinking.
6. Physical Activity: defined as sufficient if participants reported ≥150 min/week of moderate-intensity activity, ≥75 min/week of vigorous-intensity activity, or an equivalent combination of moderate- and vigorous-intensity activity. Participants below these thresholds were classified as insufficiently active (10).
7. Vegetable Intake: assessed separately from fruit and defined as insufficient (<400 g/day) or sufficient (≥400 g/day) (11).
8. Red Meat Intake: categorized as insufficient (<350 g/week), moderate (350–500 g/week), or excessive (≥500 g/week) (12).
9. Sun Exposure Frequency: assessed as self-reported average daily outdoor sun exposure and categorized as <2 h/day, 2–8 h/day, or >8 h/day. These categories were derived from the routine health examination questionnaire to distinguish limited, moderate, and prolonged daily sun exposure. Self-reported time outdoors has been commonly used in epidemiological assessment of sun exposure, although no universally accepted cutoff exists for cardiometabolic research (13).
10. Tea Consumption: categorized as none, fully fermented tea (black tea), semi-fermented tea (oolong tea), or unfermented tea (green tea) (14).
11. Average Sleep Duration: categorized as short (<5 h/day), moderate (5–7 h/day), or long (≥7 h/day) (15).
12. Sedentary behavior: assessed as self-reported daily sitting time and categorized as <4 h/day, 4–<8 h/day, or ≥8 h/day. The ≥8 h/day category was used to indicate prolonged sedentary exposure, consistent with sedentary-behavior terminology and adult movement-behavior guidance that use approximately 8 h/day as a high sedentary-time threshold (16, 17).

### 2.5 Statistical analysis

Descriptive statistics and primary multivariable analyses were conducted using the complete-case sample (*n* = 11,291). Continuous variables were presented as medians with interquartile ranges because of non-normal distributions and were compared using the Mann–Whitney U test. Categorical variables were presented as frequencies and percentages and were compared using the chi-square test. Stratified prevalence estimates of MetS were reported with 95% confidence intervals (CIs), and trend tests were used for ordinal variables where appropriate.

Primary multivariable logistic regression models were fitted in the complete-case sample to estimate odds ratios and 95% confidence intervals for associations between lifestyle factors and MetS. For each exposure, exposure-specific models were adjusted for sex, age group, annual household income, and prespecified lifestyle covariates, as detailed in Table 1. BMI was not included in the main models because central obesity is embedded in the IDF definition of MetS and BMI is closely related to adiposity.

**Table 1:** Associations between lifestyle factors and metabolic syndrome in the primary complete-case multivariable models (*n* = 11,291).

Exposure	Level	OR (95% CI)	*p*-value
Physical activity	Sufficient/active	0.85 (0.747–0.955)	0.007
Alcohol consumption	Current	1.40 (1.222–1.602)	<0.001
Smoking	Current/former (≥1 year)	1.21 (1.063–1.369)	0.004
Red meat intake	Insufficient	0.81 (0.653–1.011)	0.0627
Red meat intake	Moderate	0.85 (0.756–0.955)	0.006
Vegetable consumption	Sufficient	1.00 (0.872–1.138)	0.9566
Average sleep duration	5–<7 h/day	0.98 (0.804–1.190)	0.8242
Average sleep duration	≥7 h/day	0.91 (0.726–1.128)	0.3755
Sedentary behavior	≥8 h/day	0.91 (0.756–1.088)	0.2914
Sedentary behavior	4–<8 h/day	1.00 (0.866–1.161)	0.9734
Sun exposure	2–8 h/day	1.23 (1.083–1.388)	0.001
Sun exposure	>8 h/day	1.56 (0.997–2.452)	0.0517
Tea consumption	Fully fermented	1.20 (0.974–1.473)	0.0865
Tea consumption	Unfermented	1.44 (1.153–1.803)	0.001
Tea consumption	Semi-fermented	1.29 (0.886–1.875)	0.1842

Data are presented as odds ratios (ORs) with 95% confidence intervals (CIs) from exposure-specific logistic regression models in the complete-case sample. For each exposure, the model adjusted for sex, age group, annual household income, and prespecified exposure-specific lifestyle covariates. BMI was not included in the main model because central obesity is embedded in the IDF definition of metabolic syndrome. Reference categories were insufficient physical activity, non-drinking, non-smoking, excessive red meat intake, insufficient vegetable consumption, sleep duration <5 h/day, sitting time <4 h/day, sun exposure <2 h/day, and no tea consumption.

Among the 22,156 eligible participants, 10,865 (49.0%) had incomplete biochemical and/or questionnaire data. Because excluded eligible examinees differed from included participants in several baseline characteristics, multiple imputation by chained equations was performed as a sensitivity analysis using the full eligible cohort. Thirty imputed datasets were generated using the mice package in R. The imputation model included the outcome variable, all variables used in the regression models, and auxiliary cardiometabolic variables including waist circumference and blood pressure measurements. Estimates were pooled using Rubin’s rules.

An additional fully adjusted BMI-adjusted sensitivity model was fitted within the imputed datasets, including BMI, sex, age group, annual household income, and all lifestyle variables simultaneously. This model was used to assess whether the main lifestyle associations, particularly physical activity, were robust after further adjustment for adiposity and mutual adjustment for other lifestyle factors.

Multicollinearity was assessed using variance inflation factors (VIFs), with VIF values below 4.0 considered to indicate no serious multicollinearity. All analyses were conducted in R using the mice, dplyr, and broom packages. A two-sided *p*-value <0.05 was considered statistically significant.

## 3 Results

### 3.1 Baseline characteristics and prevalence of metabolic syndrome

**Figure 1:**
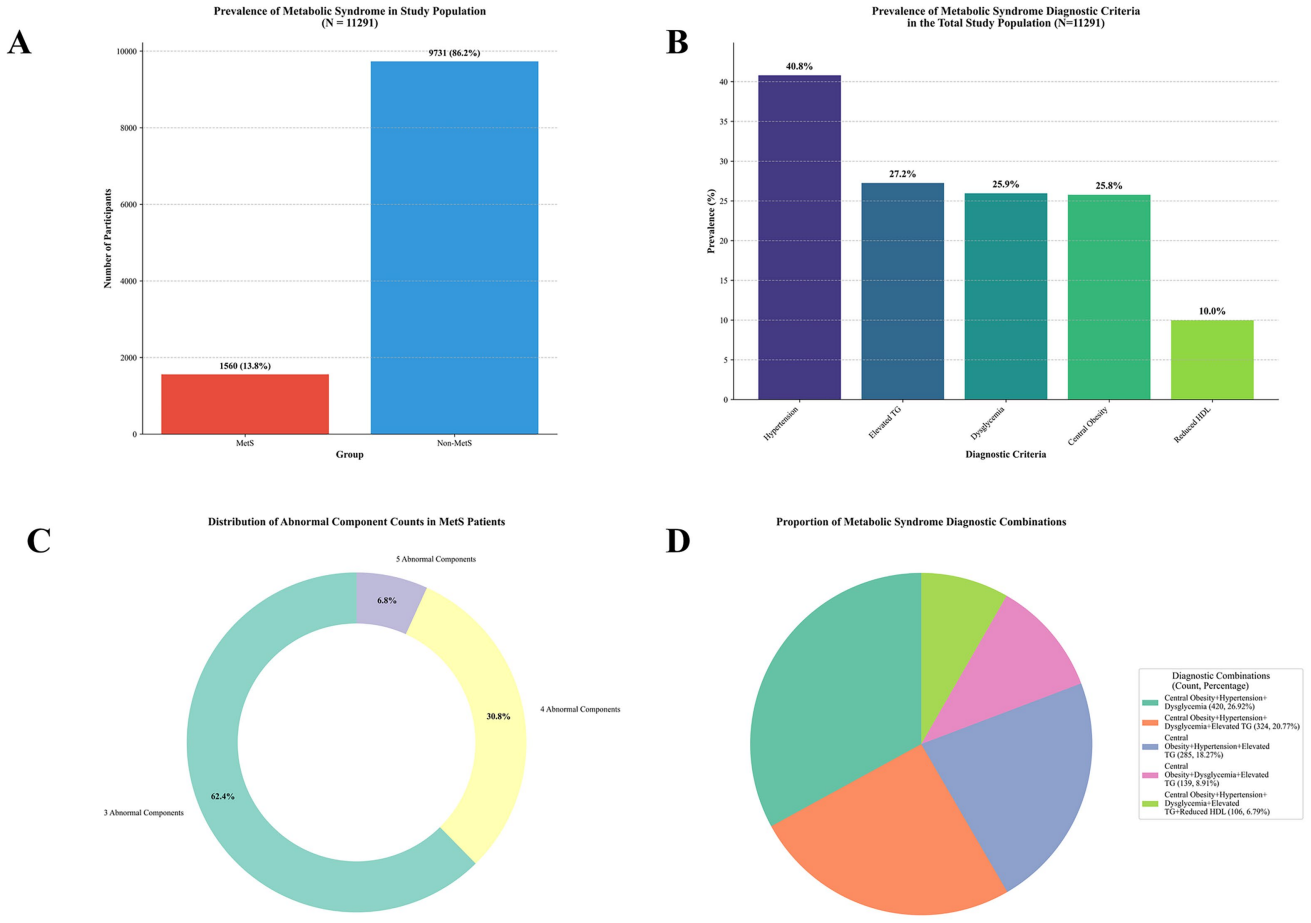
Prevalence and distribution of metabolic syndrome and its components in the complete-case study population. **(A)** Overall prevalence of MetS. **(B)** Prevalence of individual diagnostic components. **(C)** Distribution of abnormal component counts among participants with MetS. **(D)** Proportions of common diagnostic combinations.

Of the 11,291 participants with complete data, 1,560 (13.8%) met the IDF criteria for MetS (Table 2; Figure 1A). At the component level, elevated blood pressure was the most prevalent abnormality (40.8%), followed by elevated triglycerides (27.2%), elevated fasting glucose (25.9%), central obesity (25.8%), and reduced HDL-C (10.0%; Figure 1B).

**Table 2:** Baseline demographic, anthropometric, and lifestyle characteristics by metabolic syndrome status (*n* = 11,291).

Variable	Total (*n* = 11,291)	No MetS (*n* = 9,731)	With MetS (*n* = 1,560)	*p*-value
Sex				<0.001
Female	3,618 (32.0)	3,391 (34.8)	227 (14.6)	
Male	7,673 (68.0)	6,340 (65.2)	1,333 (85.4)	
Age, years	48.0 (39.0–57.0)	47.0 (38.0–56.0)	54.0 (47.0–63.0)	<0.001
BMI, kg/m^2^	24.1 (22.1–26.2)	23.6 (21.7–25.4)	27.7 (26.3–29.4)	<0.001
Waist Circumference, cm	83.0 (76.0–89.0)	81.0 (75.0–86.0)	94.0 (91.0–98.0)	<0.001
Systolic BP, mmHg	120.0 (110.0–132.0)	118.0 (109.0–129.0)	134.0 (125.0–142.0)	<0.001
Diastolic BP, mmHg	71.0 (64.0–79.0)	70.0 (63.0–77.0)	79.0 (71.8–86.0)	<0.001
HDL-C, mmol/L	1.4 (1.2–1.7)	1.4 (1.2–1.7)	1.2 (1.1–1.4)	<0.001
Triglycerides, mmol/L	1.2 (0.8–1.8)	1.1 (0.8–1.6)	2.0 (1.5–2.8)	<0.001
LDL-C, mmol/L	2.9 (2.4–3.5)	2.9 (2.4–3.4)	2.9 (2.3–3.5)	0.38
Fasting Glucose, mmol/L	5.2 (4.8–5.5)	5.1 (4.8–5.4)	5.8 (5.3–6.3)	<0.001
Education level				<0.001
Low	121 (1.1)	85 (0.9)	36 (2.3)	
Moderate	511 (4.5)	392 (4.0)	119 (7.6)	
High	10,659 (94.4)	9,254 (95.1)	1,405 (90.1)	
Annual income				<0.001
Low	936 (8.3)	734 (7.5)	202 (12.9)	
Moderate	5,708 (50.6)	4,910 (50.5)	798 (51.2)	
High	3,452 (30.6)	3,030 (31.1)	422 (27.1)	
Very high	1,195 (10.6)	1,057 (10.9)	138 (8.8)	
Sun exposure frequency				<0.001
<2 h/day	8,560 (75.8)	7,492 (76.9)	1,068 (68.5)	
2–8 h/day	2,597 (23.0)	2,131 (21.9)	466 (29.9)	
>8 h/day	134 (1.2)	108 (1.1)	26 (1.7)	
Smoking status				<0.001
Never	8,715 (77.2)	7,693 (79.1)	1,022 (65.5)	
Current/Former (≥1 year)	2,576 (22.8)	2,038 (20.9)	538 (34.5)	
Alcohol consumption				<0.001
Never	9,367 (83.0)	8,214 (84.4)	1,153 (73.9)	
Current	1,924 (17.0)	1,517 (15.6)	407 (26.1)	
Tea consumption				<0.001
None	1,321 (11.7)	1,200 (12.3)	121 (7.8)	
Fully-Fermented	7,057 (62.5)	6,072 (62.4)	985 (63.1)	
Semi-Fermented	327 (2.9)	280 (2.9)	47 (3.0)	
Unfermented	2,586 (22.9)	2,179 (22.4)	407 (26.1)	
Average sleep duration				0.003
<5 h	869 (7.7)	720 (7.4)	149 (9.6)	
5–7 h	7,733 (68.5)	6,661 (68.5)	1,072 (68.7)	
≥7 h	2,689 (23.8)	2,350 (24.1)	339 (21.7)	

Data are presented as median (interquartile range) or n (%), as appropriate. p-values were calculated using the Mann–Whitney U test for continuous variables and the chi-square test for categorical variables. BMI, body mass index; HDL-C, high-density lipoprotein cholesterol; LDL-C, low-density lipoprotein cholesterol.

Among participants with MetS, 62.4% had three abnormal components, 30.8% had four, and 6.8% had all five abnormalities (Figure 1C). The most common diagnostic pattern was central obesity plus elevated blood pressure and hypertriglyceridemia (26.9%), followed by central obesity plus elevated blood pressure, hypertriglyceridemia, and hyperglycemia (20.8%; Figure 1D).

### 3.2 Stratified prevalence of metabolic syndrome

The overall prevalence of MetS was 13.8% in the complete-case sample. Stratified analyses showed marked differences across demographic, anthropometric, and lifestyle subgroups (Table 3). Men had a higher prevalence than women (17.4% vs. 6.3%, *p* < 0.001). Prevalence increased progressively with age, ranging from 5.1% among participants aged <40 years to 24.1% among those aged ≥ 60 years (P-trend <0.001).

**Table 3:** Stratified prevalence of metabolic syndrome by demographic, anthropometric, and lifestyle factors (*n* = 11,291).

Variable	Category	n/N	Prevalence,% (95% CI)	*p*
Overall	—	1,560/11,291	13.8 (13.2–14.5)	—
Sex	Female	227/3,618	6.3 (5.5–7.1)	<0.001
	Male	1,333/7,673	17.4 (16.5–18.2)	
Age group, years	<40	155/3,036	5.1 (4.4–5.9)	<0.001†
	40–49	361/3,053	11.8 (10.7–13.0)	
	50–59	520/3,025	17.2 (15.9–18.6)	
	≥60	524/2,177	24.1 (22.3–25.9)	
BMI category	18.5–23.9 (Normal)	52/5,329	1.0 (0.7–1.3)	<0.001†
	24.0–27.9(Overweight)	777/4,607	16.9 (15.8–18.0)	
	≥28.0 (Obese)	731/1,355	53.9 (51.3–56.6)	
Blood pressure status	Normal	247/5,375	4.6 (4.1–5.2)	<0.001†
	Elevated	1,011/5,039	20.1 (19.0–21.2)	
	Hypertension	302/877	34.4 (31.4–37.6)	
Education level	Low	36/121	29.8 (22.3–38.4)	<0.001†
	Moderate	119/511	23.3 (19.8–27.1)	
	High	1,405/10,659	13.2 (12.6–13.8)	
Annual income	Low	202/936	21.6 (19.1–24.3)	<0.001†
	Moderate	798/5,708	14.0 (13.1–14.9)	
	High	422/3,452	12.2 (11.2–13.4)	
	Very High	138/1,195	11.5 (9.9–13.5)	
Tea consumption	Never	121/1,321	9.2 (7.7–10.8)	<0.001
	Fully Fermented	985/7,057	14.0 (13.2–14.8)	
	Semi-Fermented	47/327	14.4 (11.0–18.6)	
	Unfermented	407/2,586	15.7 (14.4–17.2)	
Sun exposure	<2 h/day	1,068/8,560	12.5 (11.8–13.2)	<0.001†
	2–8 h/day	466/2,597	17.9 (16.4–19.6)	
	>8 h/day	26/134	19.4 (13.6–26.9)	
Vegetable intake	Insufficient	1,162/8,842	13.1 (12.5–13.9)	0.001
	Sufficient	398/2,449	16.3 (14.8–17.8)	
Red meat intake	Insufficient	116/863	13.4 (11.3–15.9)	0.441
	Moderate	642/4,793	13.4 (12.5–14.4)	
	Excessive	802/5,635	14.2 (13.3–15.2)	
Sleep duration	<5 h	149/869	17.1 (14.8–19.8)	0.003†
	5–7 h	1,072/7,733	13.9 (13.1–14.7)	
	≥7 h	339/2,689	12.6 (11.4–13.9)	

CI, confidence interval. *p*-values from chi-square tests (non-ordinal variables) or Cochran-Armitage trend tests (†, ordinal variables).

Obesity showed the strongest gradient: MetS prevalence increased from 1.0% in normal-weight participants to 16.9% in overweight participants and 53.9% in obese participants (P for trend <0.001). Similarly, prevalence increased with blood pressure status, from 4.6% among participants with normal blood pressure to 34.4% among those with hypertension.

Socioeconomic and lifestyle factors also showed significant unadjusted associations. Participants with lower education and income levels had higher MetS prevalence than those with higher socioeconomic status. Current/former smoking, current alcohol consumption, and short sleep duration were associated with higher MetS prevalence. Higher sun exposure and unfermented tea consumption were also associated with higher prevalence in unadjusted analyses.

### 3.3 Multivariable logistic regression analysis of factors associated with metabolic syndrome

Table 1 presents the primary complete-case multivariable logistic regression results (*n* = 11,291). Sufficient physical activity was inversely associated with MetS (OR = 0.85, 95% CI: 0.75–0.96, *p* = 0.007). Current alcohol consumption (OR = 1.40, 95% CI: 1.22–1.60, *p* < 0.001) and current/former smoking (OR = 1.21, 95% CI: 1.06–1.37, *p* = 0.004) were positively associated with MetS. Sun exposure of 2–8 h/day was also associated with higher odds of MetS (OR = 1.23, 95% CI: 1.08–1.39, *p* = 0.001), whereas the >8 h/day category showed a borderline association (OR = 1.56, 95% CI: 1.00–2.45, *p* = 0.052).

Moderate red meat intake (350–<500 g/week) was inversely associated with MetS compared with excessive intake (≥500 g/week) in the primary complete-case model (OR = 0.85, 95% CI: 0.76–0.96, *p* = 0.006), whereas insufficient intake was not statistically significant. Unfermented tea consumption showed a positive association with MetS (OR = 1.44, 95% CI: 1.15–1.80, *p* = 0.001). Vegetable consumption, sleep duration, and sedentary behavior were not significantly associated with MetS.

In the multiple-imputation sensitivity analysis using the full eligible cohort, sufficient physical activity remained inversely associated with MetS (OR = 0.80, 95% CI: 0.72–0.89, *p* < 0.001), while smoking was not statistically significant (Supplementary Table S2). In the fully adjusted MI-based BMI-adjusted sensitivity analysis, BMI was strongly associated with MetS (OR = 1.78 per 1 kg/m^2^ increase, 95% CI: 1.74–1.81, *p* < 0.001), and sufficient physical activity remained inversely associated with MetS after additional adjustment for BMI and mutual adjustment for other lifestyle variables (OR = 0.80, 95% CI: 0.70–0.91, *p* < 0.001). By contrast, alcohol consumption, smoking, red meat intake, vegetable consumption, sleep duration, sedentary behavior, sun exposure, and tea consumption were not statistically significant in this fully adjusted sensitivity model (Supplementary Table S3). Multicollinearity diagnostics showed no evidence of serious collinearity, with all VIFs below 4.0 (Supplementary Table S4). Because different lifestyle factors were modeled using prespecified exposure-specific covariate sets, ORs should be interpreted as within-model associations rather than directly comparable effect sizes across exposures.

## 4 Discussion

This cross-sectional study of 22,156 civil servants undergoing routine health examinations in Guangzhou found that MetS was common in this selected occupational cohort. In the complete-case sample, MetS prevalence was 13.8%, with clear gradients by sex, age, and adiposity. In the primary complete-case analysis, sufficient physical activity was inversely associated with MetS, and this association was consistent in the multiple-imputation and BMI-adjusted sensitivity analyses. By contrast, several other lifestyle associations observed in the primary model were attenuated in the fully adjusted BMI-adjusted sensitivity model. These findings suggest that physical activity was the most robust lifestyle correlate of MetS in this study population.

Our findings are broadly consistent with regional patterns in southern China but show a lower MetS prevalence than many national estimates. A meta-analysis of studies from 2005 to 2015 reported a pooled prevalence of 24.5% (95% CI, 22.0–26.9%) among Chinese adults aged ≥15 years (18). Recent national and regional surveys have also reported substantial cardiometabolic burden in Chinese adults (19). The relatively lower prevalence in our cohort may reflect the selected nature of the study population, which consisted mainly of urban civil servants and professional workers undergoing routine health examinations. The very high proportion of participants with higher education also underscores the selected nature of this cohort (20). In contrast, studies of office workers and urban occupational populations have reported higher prevalence estimates, particularly among men (21, 22).

The marked differences in MetS prevalence by sex and age are consistent with previous epidemiological evidence. Men had a substantially higher prevalence than women, which may be related to greater visceral fat accumulation and associated insulin resistance (23). The age-related increase in MetS prevalence likely reflects cumulative metabolic dysregulation, including changes in insulin sensitivity, vascular function, and body composition (24). Elevated blood pressure was the most frequent abnormal component in this cohort, suggesting that blood pressure control may be an important entry point for cardiometabolic risk management in this occupational population. Although central obesity was not the most prevalent individual abnormality, it remains a prerequisite in the IDF definition of MetS and a key pathophysiological driver of metabolic dysregulation (25, 26). The expansion of adipose tissue in central obesity may lead to adipocyte dysfunction, and proinflammatory cytokines such as TNF-*α* and IL-6 released from dysfunctional adipocytes can impair insulin signaling, contributing to hypertension and hyperglycemia (27).

Among the lifestyle factors examined, physical activity showed the strongest and most consistent inverse association with MetS. This supports findings from the China Health and Nutrition Survey, where moderate activity was associated with lower MetS risks in sedentary adults (8, 28). Exercise enhances insulin sensitivity, reduces visceral fat, and modulates inflammation, and may represent an important correlate to prioritize in future occupational health research (29, 30).

In contrast, associations for alcohol consumption, sun exposure, red meat intake, and tea consumption were attenuated in the fully adjusted BMI-adjusted sensitivity model, which additionally accounted for adiposity and mutual adjustment among lifestyle factors. These findings suggest that some lifestyle associations observed in the primary complete-case model may partly reflect differences in adiposity or residual socioeconomic and behavioral confounding. Therefore, these associations should be interpreted cautiously and not considered robust independent correlates in this study.

These results have important implications for public health in rapidly urbanizing regions like the Greater Bay Area. Workplace interventions targeting sedentary professions, such as promoting ≥150 min of moderate activity weekly, may help improve cardiometabolic risk profiles and should be evaluated in future intervention studies (29). Early screening integrating anthropometric and biochemical markers is recommended, especially for older males.

Several limitations should be considered. First, the cross-sectional design precludes assessment of temporality; therefore, the observed association between physical activity and MetS should be interpreted as an association rather than direct evidence of prevention. Longitudinal and workplace-based intervention studies are needed to confirm whether increasing physical activity can reduce incident MetS in similar occupational populations. Second, 10,865 of 22,156 eligible participants had incomplete biochemical and/or questionnaire data. Although multiple imputation was used to reduce potential selection bias and improve statistical efficiency, this approach relies on the missing-at-random assumption, and residual bias cannot be fully excluded. Third, physical activity and dietary variables were self-reported, which may introduce recall and reporting bias. Finally, the single-center occupational sample limits generalizability to the broader population, and the healthy worker effect may have contributed to the relatively low observed MetS prevalence.

## 5 Conclusion

In this selected routine-examination cohort of civil servants in Guangzhou, MetS remained common, with clear gradients by sex, age, and adiposity. Among the lifestyle factors examined, physical activity was the most consistent correlate of MetS in the primary complete-case analysis and in multiple-imputation sensitivity analyses. These findings support workplace-based physical activity promotion and cardiometabolic risk management as potential priorities in occupational health programs for similar office-based populations.

## Data Availability

The datasets presented in this article are not readily available because the original data analyzed in this study are not publicly available due to institutional data protection policies and privacy restrictions related to routine health examination records. Requests to access the data may be directed to the corresponding author and will be considered subject to institutional approval. Requests to access the datasets should be directed to Wenwei Luo, luo_wenwei99@163.com.
