# Supplementary material for: Prevalence, component patterns, and lifestyle correlates of metabolic syndrome among civil servants undergoing routine health examinations in Guangzhou, China: a cross-sectional study
Source: Front Public Health. 2026 Jul 6;14:1893244. doi: 10.3389/fpubh.2026.1893244 (PMC13381463; doi:10.3389/fpubh.2026.1893244)
Supplement: Supplementary file 1 [file Table_1.docx]

Supplementary Table S1. Comparison of baseline characteristics between included participants and excluded eligible examinees.

| **Variable** | **Category** | **Overall (N=22,156)** | **Excluded eligible examinees (N=10,865)** | **Included participants (N=11,291)** | **SMD** | **P-value** |
| --- | --- | --- | --- | --- | --- | --- |
| Sex | Female | 7,607 (34.3) | 3,989 (36.7) | 3,618 (32.0) | 0.098 | <0.001 |
|  | Male | 14,549 (65.7) | 6,876 (63.3) | 7,673 (68.0) |  |  |
| Age, years |  | 51.0 [41.0, 60.0] | 54.0 [43.0, 64.0] | 48.0 [39.0, 57.0] | 0.388 | <0.001 |
| BMI, kg/m² |  | 24.1 [22.0, 26.2] | 24.0 [21.9, 26.1] | 24.1 [22.1, 26.2] | 0.038 | 0.078 |
| Waist circumference, cm |  | 83.0 [76.0, 89.0] | 83.0 [76.0, 90.0] | 83.0 [76.0, 89.0] | 0.046 | 0.011 |
| SBP, mmHg |  | 122.0 [111.0, 134.0] | 123.0 [112.0, 135.0] | 120.0 [110.0, 132.0] | 0.174 | <0.001 |
| DBP, mmHg |  | 71.0 [64.0, 79.0] | 72.0 [64.0, 79.0] | 71.0 [64.0, 79.0] | 0.041 | 0.013 |
| HDL-C, mmol/L |  | 1.4 [1.2, 1.7] | 1.4 [1.2, 1.7] | 1.4 [1.2, 1.7] | 0.074 | <0.001 |
| Triglycerides, mmol/L |  | 1.2 [0.9, 1.8] | 1.2 [0.9, 1.8] | 1.2 [0.8, 1.8] | 0.006 | 1.000 |
| LDL-C, mmol/L |  | 2.9 [2.4, 3.5] | 2.9 [2.4, 3.5] | 2.9 [2.4, 3.5] | 0.020 | 1.000 |
| FPG, mmol/L |  | 5.2 [4.9, 5.6] | 5.2 [4.9, 5.7] | 5.2 [4.8, 5.5] | 0.133 | <0.001 |
| Education level | Low | 208 (0.9) | 87 (0.8) | 121 (1.1) | 2.092 | <0.001 |
|  | Moderate | 770 (3.5) | 259 (2.4) | 511 (4.5) |  |  |
|  | High | 13,751 (62.1) | 3,092 (28.5) | 10,659 (94.4) |  |  |
|  | Missing | 7,427 (33.5) | 7,427 (68.4) | 0 (0.0) |  |  |
| Annual household income | Low | 1,288 (5.8) | 352 (3.2) | 936 (8.3) | 2.346 | <0.001 |
|  | Moderate | 7,268 (32.8) | 1,560 (14.4) | 5,708 (50.6) |  |  |
|  | High | 4,237 (19.1) | 785 (7.2) | 3,452 (30.6) |  |  |
|  | Very High | 1,483 (6.7) | 288 (2.7) | 1,195 (10.6) |  |  |
|  | Missing | 7,880 (35.6) | 7,880 (72.5) | 0 (0.0) |  |  |
| Alcohol consumption | Never | 12,628 (57.0) | 3,261 (30.0) | 9,367 (83.0) | 2.036 | <0.001 |
|  | Current | 2,245 (10.1) | 321 (3.0) | 1,924 (17.0) |  |  |
|  | Missing | 7,283 (32.9) | 7,283 (67.0) | 0 (0.0) |  |  |
| Sleep duration | <5 h/day | 1,114 (5.0) | 245 (2.3) | 869 (7.7) | 2.261 | <0.001 |
|  | 5-7 h/day | 9,757 (44.0) | 2,024 (18.6) | 7,733 (68.5) |  |  |
|  | ≥7 h/day | 3,497 (15.8) | 808 (7.4) | 2,689 (23.8) |  |  |
|  | Missing | 7,788 (35.2) | 7,788 (71.7) | 0 (0.0) |  |  |

Note: Data are presented as n (%) or median [interquartile range], as appropriate. Excluded eligible examinees were those excluded from the final analytic sample because of incomplete biochemical and/or questionnaire data. Group differences were compared using the chi-square test or Mann–Whitney U test, as appropriate. Standardized mean differences (SMDs) are shown to reflect the magnitude of between-group differences. Missing categories were displayed explicitly for questionnaire-based variables because incomplete questionnaire data contributed to exclusion.
