# Supplementary material for: Prevalence, component patterns, and lifestyle correlates of metabolic syndrome among civil servants undergoing routine health examinations in Guangzhou, China: a cross-sectional study
Source: Front Public Health. 2026 Jul 6;14:1893244. doi: 10.3389/fpubh.2026.1893244 (PMC13381463; doi:10.3389/fpubh.2026.1893244)
Supplement: Supplementary file 3 [file Table_3.docx]

Supplementary Table S3. Fully adjusted BMI-adjusted sensitivity analysis after multiple imputation

| **Exposure** | **Level** | **OR** | **95% CI** | **P-value** |
| --- | --- | --- | --- | --- |
| BMI | Per 1 kg/m² increase | 1.775 | 1.739–1.812 | <0.001 |
| Physical activity | Sufficient / active | 0.800 | 0.702–0.910 | <0.001 |
| Alcohol consumption | Current | 1.152 | 0.992–1.338 | 0.064 |
| Smoking | Current/former | 1.005 | 0.888–1.138 | 0.936 |
| Red meat intake | Insufficient | 1.054 | 0.852–1.306 | 0.626 |
|  | Moderate | 1.016 | 0.905–1.140 | 0.791 |
| Vegetable consumption | Sufficient | 0.968 | 0.864–1.084 | 0.572 |
| Average sleep duration | >7 hours/day | 1.065 | 0.933–1.217 | 0.350 |
| Sedentary behavior | 4–8 h/day | 1.113 | 0.967–1.280 | 0.135 |
| Sun exposure | 2–8 h/day | 1.129 | 0.869–1.467 | 0.364 |
|  | >8 h/day | 1.439 | 0.929–2.229 | 0.102 |
| Tea consumption | Fully fermented | 0.926 | 0.800–1.071 | 0.298 |
|  | Semi-fermented | 0.814 | 0.658–1.006 | 0.057 |
|  | Unfermented | 1.083 | 0.901–1.301 | 0.392 |

Note: Data are presented as odds ratios (ORs), 95% confidence intervals (CIs), and P-values from a fully adjusted BMI-adjusted logistic regression model after multiple imputation. Thirty imputed datasets were generated and estimates were pooled using Rubin’s rules. The model included BMI, sex, age group, annual household income, physical activity, alcohol consumption, smoking status, red meat intake, vegetable consumption, average sleep duration, sedentary behavior, sun exposure, and tea consumption simultaneously. BMI was included only in this sensitivity analysis because central obesity is embedded in the IDF definition of metabolic syndrome.Reference categories were male sex, age <40 years, high annual household income, insufficient physical activity, non-drinking, non-smoking, excessive red meat intake, insufficient vegetable consumption, sleep duration 5–7 h/day, sitting time <4 h/day, sun exposure <2 h/day, and no tea consumption.
