# Supplementary material for: Prevalence, component patterns, and lifestyle correlates of metabolic syndrome among civil servants undergoing routine health examinations in Guangzhou, China: a cross-sectional study
Source: Front Public Health. 2026 Jul 6;14:1893244. doi: 10.3389/fpubh.2026.1893244 (PMC13381463; doi:10.3389/fpubh.2026.1893244)
Supplement: Supplementary file 4 [file Table_4.docx]

Supplementary Table S4.Variance inflation factors

| **Variable** | **VIF** |
| --- | --- |
| annual_household_income_moderate | 3.730 |
| annual_household_income_high | 3.638 |
| average_sleep_duration_>7 hours | 3.233 |
| average_sleep_duration_5.1–7 hours | 3.205 |
| daily_sitting_time_>8 h/day | 2.451 |
| annual_household_income_very high | 2.248 |
| daily_sitting_time_4–8 h/day | 2.203 |
| age_group_>=60 | 1.737 |
| age_group_50–59 | 1.608 |
| age_group_40–49 | 1.529 |
| Gender_Male | 1.218 |
| years_of_smoking_yes | 1.197 |
| alcohol_consumption_status_yes | 1.122 |
| physical_activity_yes | 1.034 |

Variance inflation factors (VIFs) were calculated for the main multivariable model to assess multicollinearity. No serious multicollinearity was observed (all VIFs <4). Values shown below are from the main model for physical activity, which used the same analytic sample and adjustment framework as the primary results. Sedentary behavior was measured in hours per day (h/day).
