# Supplementary material for: Prevalence, component patterns, and lifestyle correlates of metabolic syndrome among civil servants undergoing routine health examinations in Guangzhou, China: a cross-sectional study
Source: Front Public Health. 2026 Jul 6;14:1893244. doi: 10.3389/fpubh.2026.1893244 (PMC13381463; doi:10.3389/fpubh.2026.1893244)
Supplement: Supplementary file 5 [file Table_5.docx]

**Supplementary Table S5. Questionnaire-derived variables used in the analysis**

Note. Items are English translations of the original Chinese electronic questionnaire used in the routine health examination program. Original response options are retained where relevant, and all recoding, category collapsing, and unit conversion used in the present analysis are explicitly described. Most questionnaire-derived variables were single-item or item-group screening questions rather than multi-item psychometric scales; therefore, subscales and internal consistency measures such as Cronbach's alpha were not applicable. One liang was treated as 50 g for unit conversion. Abbreviation: h, hours.

| **Domain** | **Variable used in analysis** | **No. of source item(s)** | **Questionnaire item / source question** | **Original response options** | **Recoding or unit conversion** | **Analytic coding in this study** |
| --- | --- | --- | --- | --- | --- | --- |
| Sociodemographic characteristics | Education level | 1 | Q8. What is your highest level of education? | Below primary school; junior high school; high school or vocational school; associate or bachelor's degree; master's degree or above. | Collapsed into three analytic categories. | Low = below primary school or junior high school; moderate = high school or vocational school; high = associate/bachelor's degree or master's degree or above. |
| Sociodemographic characteristics | Annual household income | 1 | Q6. What is your annual household income? | <100,000 RMB; 100,000-300,000 RMB; 300,000-500,000 RMB; >500,000 RMB. | Original response categories were retained and relabeled for analysis. | Low = <100,000 RMB; moderate = 100,000-299,999 RMB; high = 300,000-499,999 RMB; very high = >=500,000 RMB. |
| Environmental exposure | Sun exposure frequency | 1 | Q12. How often are you exposed to sunlight? | Never; <2 h/day; 2-4 h/day; 4-8 h/day; >8 h/day. | Original categories were collapsed to distinguish limited, moderate, and prolonged daily sun exposure. | Never and <2 h/day were combined as <2 h/day; 2-4 and 4-8 h/day were combined as 2-8 h/day; >8 h/day was retained. |
| Smoking history | Smoking status | 4 | Q15. For how many years have you smoked? Q16. What type(s) of tobacco products do/did you use? Q17. For how many years have you quit smoking? Q17_alt. On average, how many cigarettes per day do/did you smoke? | Smoking duration in years; cigarettes/e-cigarettes/loose tobacco; quit duration in years; cigarettes/day. | Smoking history variables were summarized into a binary smoking status variable. | Never smokers were participants reporting 0 years of smoking/no tobacco use. Participants reporting a smoking history of >=1 year were classified as current/former smokers. |
| Alcohol consumption | Alcohol consumption status | 4 | Q18. How often do you consume alcohol? Q18_2. If you have drunk alcohol before, for how many years? Q18_3. If you have quit drinking, for how many years? Q19. On a typical drinking occasion, how much alcohol do you consume? | Never; <1 time/week; 1-2 times/week; 3-5 times/week; >5 times/week; drinking history years; quit years; drinking amount in liang/time. | Current drinking status was derived from reported current drinking frequency. Quantity variables were not used in the primary regression models. | Non-drinking = never/no current drinking. Current drinking = any reported current alcohol consumption frequency. |
| Beverage habits | Tea consumption | 3 | Q20. What beverage(s) do you mainly drink daily? Q22_1. What type(s) of tea do you usually drink? Q22_2. Other type(s) of tea, please specify. | Do not regularly drink tea; black tea; green tea; scented tea; oolong tea; dark tea; white tea; other tea types/free text; multiple choices allowed. | Tea types were grouped according to the major fermentation categories used in the regression models. Less frequent or multiple tea types were handled according to the predefined tea-type coding in the analytic dataset. | No tea consumption = do not regularly drink tea. Fully fermented = black tea. Semi-fermented = oolong tea. Unfermented = green tea. |
| Dietary habits | Vegetable intake | 1 | Q29. How many liang of vegetables do you eat per day? | Not every day; <2 liang; 2-<5 liang; 5-<10 liang; >=10 liang. | Vegetable intake was converted from liang/day to grams/day using 1 liang = 50 g. The analytic threshold was based on the study definition of sufficient vegetable intake. | Insufficient = <400 g/day. Sufficient = >=400 g/day. Original categorical responses were mapped to the analytic coding according to the processed questionnaire dataset after unit standardization. |
| Dietary habits | Red meat intake | 2 | Q30. On average, how much meat do you eat per day? Q31. What type(s) of meat do you usually eat? | Meat amount: no meat; <1 liang; 1-<3 liang; 3-<5 liang; >=5 liang. Meat types: red meat (pork, beef, lamb); poultry; fish; shrimp/crab/shellfish/squid; animal organs; processed meat; multiple choices allowed. | Daily meat intake was converted from liang/day to grams/day using 1 liang = 50 g and multiplied by 7 to estimate weekly intake. Red meat intake was derived among participants reporting red meat as a usual meat type. | Insufficient = <350 g/week; moderate = 350-<500 g/week; excessive = >=500 g/week, according to the study coding after unit standardization and meat-type classification. |
| Entertainment and exercise | Physical activity | 17 item group | Q37_1 to Q37_17. What entertainment and exercise activities do you usually engage in? Activity type, weekly frequency, and duration per session were recorded. | Multiple activity types, including no entertainment, light exercise, vigorous exercise, singing/playing instruments, mobile phone/TV, card games/mahjong, dining/chatting, reading/calligraphy/painting, and other free-text activities; frequency and duration were recorded when applicable. | Weekly activity time was estimated from reported frequency and duration. Moderate- and vigorous-intensity activity variables were derived from the recorded activity type and duration. | Sufficient physical activity = >=150 min/week of moderate-intensity activity, >=75 min/week of vigorous-intensity activity, or an equivalent combination. Participants below these thresholds were classified as insufficiently active. |
| Sedentary behavior and daily routine | Sedentary behavior | 1 | Q38. How many hours per day do you spend sitting, including work and leisure? | <4 h/day; 4-<8 h/day; 8-<12 h/day; >=12 h/day. | Original categories were collapsed to distinguish lower, moderate, and prolonged daily sitting time. | <4 h/day retained as <4 h/day; 4-<8 h/day coded as 4-8 h/day; 8-<12 and >=12 h/day were combined as >=8 h/day. |
| Sleep duration and quality | Average sleep duration | 1 | Q43. On average, how many hours do you sleep per night, actual sleep duration, not time in bed? | <5 h; 5-<7 h; 7-<9 h; >=9 h. | Original categories were collapsed to define short, moderate, and longer sleep duration. | Short = <5 h/day; moderate = 5-<7 h/day; long = >=7 h/day. |

Additional clarification: self-reported histories of hypertension, diabetes, and dyslipidemia, if used, were obtained from routine health examination medical history records rather than the questionnaire-derived lifestyle variables listed in this table.
